# The Changing Landscape of Water Resources Planning in England

**DOI:** 10.1007/s11269-024-04072-8

**Published:** 2025-01-16

**Authors:** Ali Leonard, Jaime Amezaga, Richard Blackwell, Elizabeth Lewis, Chris Kilsby

**Affiliations:** 1 Centre for Water, School of Engineering, Newcastle University, Newcastle Upon Tyne, UK; 2 Water Resources West, Warrington, UK; 3 School of Engineering, The University of Manchester, Manchester, UK

**Keywords:** Multi-scale, Water resources, Governance, Planning, Management, Transition, Institutional analysis, Water engineering

## Abstract

Water resources planning in England has undergone a significant transformation from a fragmented, piecemeal approach to a more strategic, multi-scale framework. This shift is a response to the pressing need for increased resilience in the face of climate change, population growth, and environmental pressures. Recognising the limitations of existing planning frameworks established during privatisation, new national, regional, company, and sub-regional frameworks have emerged to address gaps and enhance strategic planning efforts. Understanding the critical pathway dependencies, opportunities, and constraints allows reforms to be designed and implemented with a better chance of success. Several key features characterise water resources planning in England. Firstly, the systems are inherently complex and fragmented, requiring tailored approaches rather than one-size-fits-all solutions. Secondly, planning operates within a neoliberal framework emphasising economic efficiency. Thirdly, subjective concepts like risk, uncertainty, and value are managed through technical quantitative methods which can pose challenges for transparency. Fourthly, while legislation often operates in silos, there is a growing demand for more integrated planning approaches. Funding and regulatory powers play crucial roles in water resources planning. Access to capital is influenced by the institutional environment and broader economic and political factors, with government and regulators ultimately holding power over the framework. Companies, driven by the profit motive, are responsible for detailed planning and delivery, regulated by standards and reputational incentives. Public participation is framed as consumer engagement. Aligning incentives for public good with financial rewards and ensuring effective regulation are vital for the framework’s success.

## Introduction

In this paper, our purpose is to outline key features of water resources planning in England bringing in Wales where relevant. The objective is to consider these features in the context of how they evolved, identifying existing pathway dependencies. This approach positions us more effectively to assess the prospective opportunities and constraints associated with proposals aimed at enhancing planning in the future.

It is possible to distinguish five approaches to water resources management; (1) allocation of water between competing uses, (2) increase supply, (3) water transfers from areas of surplus to areas of deficit, (4) reduce demand, and (5) plan use according to supply. In tandem with ensuring sufficient water supply, a range of intrinsically interconnected objectives emerge. Salient issues include risks around drought and water shortage, resilience of supply networks, health and sanitation, environmental protection, economic cost and efficiency, carbon mitigation, issues of equity spanning both time and space, preservation of landscape and heritage, and providing amenity value. How trade-offs between competing objectives are balanced depends on the values and motivations of the decision makers and those they represent. The values and motivations of those in decision making positions have changed dramatically over time as society has evolved and developed new norms, as it faced arising challenges and responded to social, political, economic, and environmental drivers (Hassan 1985; Roberts 2000; Spar and Bebenek 2009; Taylor et al. 2009).

The task of long-term planning for resilience against risks of water scarcity is confounded by inherent subjectivity and uncertainty, often characterised as a “wicked” or “messy” problem (Grafton 2017). The uncertain nature of decision-making becomes evident in long-term planning efforts that involve future projections, notably predicting substantial deficits. The complexity arises from the intersection of various objectives, governance structures, and information dynamics. Numerous actors with responsibly over water resources planning operate within a maze of rules at different administrative levels. Additionally, the coexistence of multiple plans in parallel, coupled with perceived inadequacies in planning frameworks contributes to the challenge of effective coordination. Pathway dependencies, recognised as legacy factors influencing the pace and structure of change, emerge as a pivotal aspect. Rooted in historical influences, these dependencies often entrench the status quo serving to embed the existing institutions and power structures (Sehring 2009). Understanding their temporal influence and longevity is useful to consider how proposals for change may be constrained by or fit within the existing features of governance and decision making. These pathway dependencies may exist physically, such as through “lock in” to built infrastructure, as well socially, such as through cultural memory, traditions, and practices, and reservations to change. In this way, although wider principles and broader generalisations can offer understanding and provide guidance, any study of water resources governance will depend intrinsically on the specific time and context of each case.

We focus on public water supply in England, examining the evolution, history, and path dependency of water resources planning in this region. This case study is salient as water regulators have called on water companies to meet higher standards of supply resilience in response to growing pressures from climate change, environmental needs and growth whilst still maintaining affordability (EA 2020). New national and regional governance structures have been established with the aim of enabling better collaboration across water companies and other water abstractors to find and deliver the most efficient and robust water supply infrastructure schemes. We want to understand how and why a more strategic direction and multi-scale structure has been introduced to open discussions around evaluating its likely success.

This report begins with a brief method statement, followed by an overview of the evolution of governance categorised into six periods. Following that, features of water resources planning in England are discussed. Finally, the paper concludes with possible future directions using the analysis of the paper as starting point for further evaluation and recommendations.

## Method Statement

This study takes a qualitative approach reviewing sources from published planning and policy documents and the available literature and using insights from observations of the planning process from interviews, workshops, and through being embedded in the planning process between 2020 and 2024 at national, regional, and company levels. Twenty-seven semi-structured interviews investigating the recent changes to water resources governance structures with a particular focus on the introduction of regional planning have been conducted with twenty-one participants from across the water industry. Participants included regional planning leads (9), regulators (5), government officials (1), and water resources planners from water companies (3) and consultancies (3). Three workshops were carried out with regional planning leads exploring issues arising from the interviews and lessons learned going forward. Primary and secondary material has been analysed and categorised to provide an overview through time (Section 3) and to identify key features (Section 4) of water resources planning in England using Clement’s “Politicised” variation of Ostrom’s “Institutional Analysis Development” framework to structure the analysis (Clement 2010; Clement and Amezaga 2013; Polski and Ostrom 2017).

## Evolution of the Scale of Planning

**Fig. 1:**
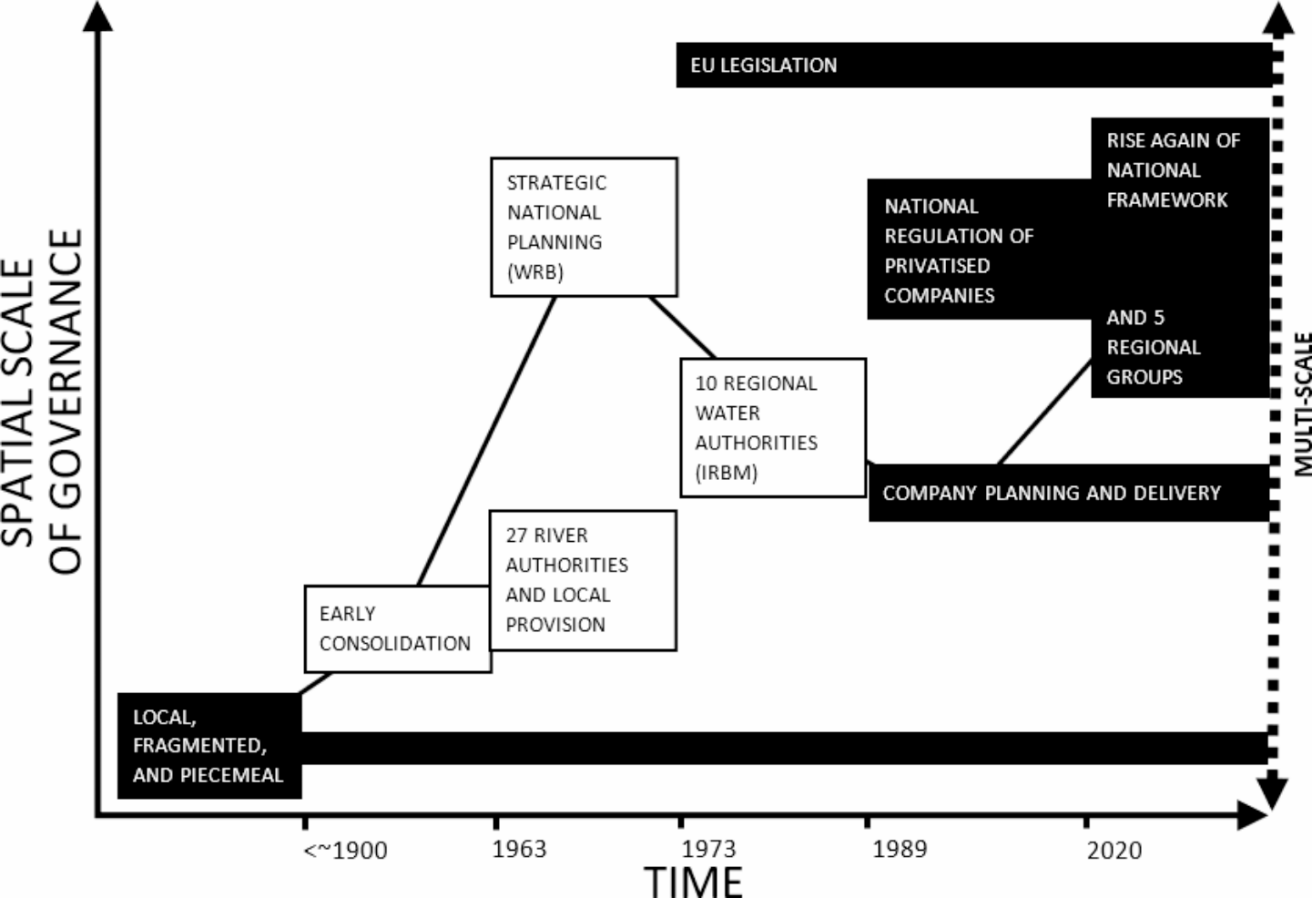
The changing scale of water resources governance in England (black filled boxes represent continuing governance structures, white filled boxes represent defunct governance structures). Acronyms: Water Resources Board (WRB), Integrated River Basin Management (IRBM)

We find that water resources planning in England has undergone several historic shifts in scale that have occurred in a non-linear fashion, responding to a complex landscape of physical and institutional pathway dependencies, drivers, and constraints. We have demarcated six periods of water resources planning; (1) piecemeal, (2) early consolidation, (3) national strategy, (4) integrated regions, (5) privatised and regulated, (6) transition to multi-scale. This evolution is illustrated in Fig. 1.

### Piecemeal

Early stages of water resources planning in England were local and fragmented. As cities and industries grew, both private enterprises and public municipalities started to develop a burgeoning water storage and supply infrastructure in a piecemeal manner to feed the rapid increase in demand for navigation, industry, workers health, and firefighting. Efforts to increase supplies were spurred further by the degradation and pollution of traditional water sources (Hassan 1985). Planning was locally driven with no overarching strategic thinking. For each new reservoir, canal, or aqueduct, companies or municipalities had to seek approval from Parliament through individual Acts (Kinnersley 1988). By the turn of the 20th century, water management was fragmented. Hundreds of local councils and private statutory companies resourced and supplied their localities. Other users, including power plants, navigation authorities, and irrigators, supplied their needs. Overall coordination was limited (Ofwat 2006).

### Early Consolidation

The second phase of water resources planning is marked by a shift in scale with greater integration and coordination to meet growing demand and tackle disparity across the rural-urban divide, for example the introduction of legislation incentivising joint working and transfers between authorities (Ofwat 2006). Alongside the advance towards universal supply and meeting growing demand from industrial users, a series of droughts occurred in the forties and fifties (Taylor et al. 2009). Reservoir development expanded exponentially to catch up with the supply and demand pressures. However, approval for new reservoir schemes faced growing opposition and placed an increasing burden on Parliament’s time. Faced with complex and contentious proposals for new infrastructure, Members of Parliament sought a new system of advice and decision making with discussions in particular continuing over the question of whether national planning should be established (Atkins 2018; McCulloch 2009).

### National Strategy

Local water supply providers continued consolidating. By 1970, statutory water undertakers were made up of 64 local authorities, 101 joint boards, and 33 statutory companies (Ofwat 2006). However, despite arguments to maintain local autonomy, a more centralised approach to planning became national policy with the introduction of the Water Resources Act, 1963. This Act, for the first-time, established organisations with specific responsibility for water resources management, conservation, and augmentation; twenty-seven newly created River Authorities and the Water Resources Board (WRB). The WRB, dominated by engineers, proposed a national water grid with new reservoirs and long-distance transfers. The top-down schemes would be funded by central bodies and aimed to control and maximise the use of water as a resource (McCulloch 2009; Smith 1997).

Unfortunately, several problems beset the Water Resource Board’s approach. Firstly, it had no jurisdiction over water quality. Against a background of worsening river pollution, there would be little point in transferring water unfit to abstract (McCulloch 2009). Secondly, large infrastructure projects, and reservoir schemes in particular, were receiving increasing criticism. Thirdly, the board’s extensive schemes were premised on inaccurate projections of continued growth in demand, justified by appeals to keep developing supply to protect domestic industry in the face of global competition (Archer and Marriot 1993; McCulloch 2009). The boards’ engineer-led, single focus on national water resources planning received growing criticism and a weakening mandate. Although some new infrastructure was built, several of the Water Resources Board’s proposed schemes were eventually abandoned.

### Integrated Regions

The fourth phase of water resources planning sees the fall of national planning and introduction of regional planning. In 1973, a new Water Act abolished the WRB. Another centralised body, the Central Water Policy Planning Unit was established with an advisory role over water resources planning and pollution control but it had limited influence (Ofwat 2006). The Act also abolished the twenty-seven River Authorities in favour of ten Regional Water Authorities (RWAs), also known as water boards. The RWAs borders were defined by the ten largest river basins and the remit was expanded in pursuit of a more integrated approach. Integrated river basin management (IRBM), which was being promoted at the European level, was central to the new institutions.

Although the 1973 reforms are often considered a form of nationalisation, it is important to note the reforms did not extend to private statutory companies which represented about twenty per cent of supply (Page and Bakker 2005).

Several interrelated problems hampered the RWAs from the outset. Their integrated nature produced an inherent tension and lack of effective scrutiny and accountability and the institutions were also hampered by a lack of finance exacerbated by a period of high inflation and instability in the wider economy (Ofwat 2006). Both these factors hindered progress towards meeting environmental targets set by the European Economic Community (EEC), which the UK had joined in 1973. The targets demanded substantial investments in water treatment infrastructure. Furthermore, wider events added further pressure to the RWAs including a severe drought in 1976, and the growing likelihood of privatisation after the election of the Conservative party in 1979, amidst growing influence of neoliberal ideas that favoured market solutions over state planning.

### Privatised and Regulated

In 1989 parts of the ten RWAs became water and sewerage limited companies (Ofwat 2006). The twenty-nine existing private water-only companies continued as before. This arrangement remains largely the same today discounting some boundary changes as companies consolidated, undergoing mergers and acquisitions. Alongside the water companies, privatisation also established the other crucial actors: the regulators. Notwithstanding some restructuring, the primary water resources regulators for water resources planning have remained relatively constant: Ofwat, in charge of economic regulation, and the Environment Agency (EA) in charge of environmental regulation, who work alongside the Department for Environment, Farming, and Rural Affairs (Defra). Since privatisation, water resources planning has been strongly steered by government and regulators through legislation, policy, guidance, and ongoing coordination (Watson et al. 2009). Meanwhile, the institutional arrangements put the technical planning, delivery, and operation in the hands of private companies clearly incentivised and legally mandated to prioritise the needs of their own customers. Consequently, the scale of planning and accountability has aligned primarily to these company boundaries.

Alongside privatisation, European legislation, notably the Water Framework Directive (WFD) 2000, has been influential in defining new scales and forms of governance. The legislation has not yet been rescinded in England despite the UK’s departure from the EU in 2016. The WFD was significant in instigating river basin as well as catchment-based planning. The regional river basin approach is mandated for all EU member states and River Basin Management Plans (RBMPs) have been produced every six years since 2009. The river basin approach aimed to establish a more integrated and holistic approach to water management to meet “good status” targets by 2015, or as a backstop by 2027. The Catchment Based Approach, or CaBA, was established in 2011 by Defra (2013) as a voluntary initiative. One hundred catchments were defined with the objective to develop local partnerships, supported by EA catchment coordinators, to support meeting the “good status” objectives in tandem with RBMPs.

Calls for institutional restructuring stemmed from concerns over resilience to drought and water scarcity, propelled by the 2010–2012 drought. A new duty, the resilience objective was codified in the Water Act 2014: “to secure the long-term resilience of water undertakers’ supply systems” (Sect. 22, 2DA(a)). Later reports projected large future deficits and called for investment in water resources infrastructure to avoid high emergency costs in the future (National Infrastructure Commission 2018; Water 2016). Furthermore, despite growing acceptance of the need to invest in drought resilience, particularly in the southeast of England, several barriers were understood to block planning efforts. Firstly, the limitations of company based zonal planning, which, as described above, incentivised companies to provide for their own customers, rather than work collaboratively with other companies and third parties to identify more efficient solutions on a regional or national basis. Secondly, there was also a growing acknowledgement that the decision-making frameworks used by companies to assess and possibly demonstrate the need for large infrastructure schemes were inadequate, with no new reservoirs constructed since privatisation, and particularly following the failure to gain consent for the proposed Abingdon Reservoir in Oxfordshire in 2010, and proposed delays to the scheme in water resource management plans in 2019. The combination of these factors (need to increase resilience, projected deficits, lack of collaboration across companies and regulators to identify the most efficient solutions, and inability of decision-making frameworks to implement schemes) drove new policy, legislative, and institutional changes targeted at improving national resilience to drought and water scarcity.

### Transition to Multi-scale Planning

The sixth and current phase of planning marks a transition to multi-scale planning including the return of more strategic, national planning. This is evidenced by (1) regulatory support and funding for strategic supply options, (2) nationally focused policy and legislation establishing new ambitious targets and (3) accompanying institutional changes with the establishment of regional planning to help deliver the objectives.

Firstly, to facilitate the development of strategic water supply options, Ofwat organised a new regulatory alliance in 2019 and made £469 million pounds available in funding for water companies to progress schemes. The Regulators’ Alliance for Progressing Infrastructure Development (RAPID) represents a partnership between Ofwat, the EA, and the Drinking Water Inspectorate (DWI) with the aim to facilitate construction of Strategic Resource Options (SROs) between 2025 and 2030. The initial list of options includes a variety of cross-boundary water transfers, reservoirs, and reuse schemes to be assessed alongside water company and regional planning.

Secondly, complementing the progression of nationally significant water supply infrastructure schemes, another more encompassing piece of national policy was published in 2020. The National Framework (EA 2020) set out ambitious targets to “meet national needs” through improving drought resilience and reducing pressures on the environment in recognition of climate change and growth forecasts. Targets for water companies include the introduction of a new resilience standard such that companies should plan based on 0.2% annual probability of emergency drought restrictions, such as rota cuts and standpipes, improving the previous 0.5% standard. Targets were also introduced to reduce demand per capita from an average of 150 L per day to 110 L a day and halve leakage from 2017 levels (on average around 20% of treated water is currently lost to leakage) by 2050. Furthermore, ambitions were set to reduce abstraction rates from surface and groundwater stores in line with new “environmental destination” targets.

Thirdly, the National Framework introduces new regional institutional arrangements to deliver the national objectives through encouraging collaboration across water companies and other stakeholders. The regional groups have been tasked to identify nationally efficient solutions to the resilience challenge. This may include cross-border solutions, such as water transfers and multi-stakeholder schemes, if they can be demonstrated as having greater benefits than options identified under traditional company planning. This regional tier extended existing regional governance (Water Resources South East (WRSE) since 1997 and Water Resources East (WRE) since 2014) across the rest of England with the creation of three new regions: Water Resources West (WRW) (which includes parts of Wales), Water Resources North (WReN), and West Country Water Resources (WCWR). Regional planning is now operating on a voluntary basis with final plans to be published in 2024 alongside company Water Resource Management Plans (WRMPs). Although regional planning is currently voluntary, the Environment Act 2021 tentatively gave new powers to the Secretary of State to direct “two or more water undertakers to prepare and publish a joint proposal”.

This transitional period marks an interesting turn to multi-scale planning with directives coming from the government and the regulators around meeting national needs to be carried out through the workings of five new regional groups, currently operating on a voluntary basis. These strategic approaches are working alongside continuing legally mandated and highly regulated water company planning, which has developed and matured over the last three decades. How this transition unfolds depends on whether the design and implementation of the new multi-scale planning structures and agendas are suitable. Evaluating the suitability of proposed reforms depends on a deep understanding of the context they are applied to.

Thus, the following section sets out core features of water resources planning in England, considering how they have come to be and why they are important for meeting stated objectives around improving the level of resilience against water scarcity, improving the condition of the environment, ensuring plentiful water for economic and societal needs, and providing this service within the constraints of affordable budgets.

## Features of Planning

This section details key features of water resources planning in the English and Welsh context using the politicised IAD framework to help guide the contextual analysis according to five categories (1) biophysical/material conditions, (2) political-economic context, (3) planning discourses, (4) rules, and (5) communities of actors (Clement 2010; Clement and Amezaga 2013). The boundaries between categories and features are somewhat blurred, as in many attempts at categorisations, but hopefully the analysis still provides useful context as to the evolution and operation of water resources planning in England.

### Biophysical/Material Conditions: Fragmented Boundaries

This section discusses the fragmented boundaries of water resources planning in England. There are several aspects to this heterogeneity and fragmentation: (1) spatial patterns of supply demand and existing built infrastructure, and geographies of (2) political and (3) institutional fragmentation. The complex and interconnected social, physical, and technical systems of storage, abstraction, treatment, and management may have local constraints heretical to “one-size-fits-all” assumptions that are often useful for consistency when planning but sometime encounter difficulties when put into practice.

#### Spatial patterns of supply, demand, and built infrastructure

The climate in England is wetter in the west, and drier in the east reflecting climate and topography (Browning et al. 1975). Combined with the expected north-south gradient in temperature and evaporation, this creates a wetter northwest and drier southeast (Simpson et al. 2016). Such a pattern is mirrored in the use of surface or groundwater, with greater reliance on surface water stores in the north and west and greater reliance on groundwater stores in the south and east, partly reflecting the varied hydro-geology of the UK (Abesser and Lewis 2015).

The pattern of water demand is also geographically fragmented and continuously evolving according to socio-economic trends, such as industrialisation and its decline, agricultural needs, population growth, and changing water use practices (Butler and Memon 2006). Per capita consumption is expected to decline with measures such as the roll out of smart metering, water efficient devices and public water saving campaigns, however, behaviour regarding water consumption is difficult to predict and there remains significant uncertainty over the pace, scale, and even direction of change (Butler and Memon 2006; Roberts 2000). Pockets of high demand are expectedly concentrated according to cities, particularly in the southeast and around London. Irrigation demand is concentrated in the East Anglian region. Industrial demand, once concentrated in the north and Wales, has declined with the decline of industries like coal and steel (Hassan 1985). Overall, there remains an enduring pattern of higher water demand in the south and east.

Thirdly, existing built infrastructure reflects historical investments such as into reservoirs and aqueducts. Noteworthy long distance transfers include connections from the Lake District to Manchester (Harwood, 1895; Taylor et al. 2009), from Wales to Liverpool and Birmingham (Roberts 2000), and from rural Northumberland to industrial centres along the Rivers Tyne and Tees (Soulsby et al. 1999). The rapid expansion of infrastructure initially with industrialisation and later with the WRB’s vision for a national grid has slowed down in fact to a halt, with the last reservoir constructed in 1991. In the southeast, despite debates going back to the sixties, proposals for wider connectivity, such as between the River Severn and the River Thames, were never realised. Additionally, it is important to recognise geographical fragmentation between water resources infrastructure and water quality infrastructure as limited water treatment capacity poses a difficulty for the expansion of water resources infrastructure, Overall, there has been more network integration in the north and west relative to the south (Simpson et al. 2016).

These physical characteristics are fundamental to the pattern of water supply in England setting constraints and underlying the direction of travel of proposals for transfers from the north and west towards the south and east.

#### Geographies of political fragmentation

Political fragmentation also plays a part, particularly between England and Wales. For example, a controversial case was a reservoir built in Wales to supply Liverpool, which drowned Tryweryn Valley, considered a bastion of Welsh culture and language (Atkins 2018). Liverpool City Council gained approval through Westminster avoiding a public inquiry and the need to gain consent from Welsh authorities despite almost unanimous opposition (Liverpool Corporation Act, 1957). This top-down approach exposed the Welsh MPs lack of political power in determining the future of Welsh resources and added impetus to Welsh devolution. The Tryweryn case is still remembered today more than sixty years later. This was evidenced in the House of Lords in 2019 when Lord Wigley described Tryweryn as an experience that *“colours all our considerations in Wales of issues relating to the supply of water to English conurbations*” (House of Lords, 2019) potentially indicating signs of a more tentative approach from Wales towards water sharing with England in light of the shared history.

#### Geographies of institutional fragmentation

Finally, water resources planning is fragmented by the institutional and governance arrangements of suppliers. As previously mentioned, the current governance structures for public water supply are heavily influenced by boundaries defined in 1973 which established the ten RWAs, which later became the ten privatised major water and sewage undertakers, alongside the twenty-nine smaller private companies. The twenty-nine smaller private companies were mostly based in the southeast and have since reduced in number having undergone consolidation through mergers and acquisitions (Ofwat 2006). The legacy of the small, private water-only companies has resulted in provision in the southeast remaining more fragmented compared to the rest of the country. This fragmentation acts as barrier to improving drought resilience by hindering network integration. The lack of regional connectivity in the southeast drove the establishment of the regional coordination body, WRSE, in 1997 to encourage inter-company collaboration. In contrast, the north integrated before privatisation. The three large water companies in the north, Northumbrian Water, Yorkshire Water and United Utilities inherited already connected and integrated infrastructure which, with some additional investment, enabled them to create single grids that now supply between 98 and 99% of their customers. Since privatisation, cross-company network integration has been limited, as legal requirements mandate companies to prioritise their own customers’ needs without obligations to collaborate with others beyond their own boundaries. This issue has prompted recent efforts to encourage greater cooperation between companies. It led to the introduction of national and regional planning to encourage more collaboration and network integration, as well as greater consideration of non-public water supply abstractors and other sectors (EA 2020). As discussed earlier, this included the establishment of regional groups (WRSE, WRE, WRW WReN, and WCWR). These new regional structures are redefining the boundaries of water resources planning providing platforms for greater collaboration between water companies as well as other abstractors and wider stakeholders.

With greater emphasis on regional, as well as national and catchment planning, these recent developments mark a critical shift towards multi-scale planning for which an important objective is overcoming and capitalising on the geographical fragmentation in all its forms.

### Political-Economic Context

We find ourselves within a period shaped by privatisation and regulation since the uptake of neoliberalism (Page and Bakker 2005). The decision-making space is relatively constrained within legally defined boundaries and fiduciary responsibilities. Delivery of water for all existing or future demands is mandated to be provided by private companies, motivated by profit, within a regulatory system that emphasises economic efficiency (McCulloch 2009).

The UK is relatively rare in having a privatised water supply. At privatisation, the new regulatory framework set minimum legal service standards and enabled the water companies to access capital (Bakker 2003). Investment has delivered improvements in service levels across a range of areas, including reductions in leakage, as well as improved drinking water quality, and wastewater treatment. The combination of greater focus on economic efficiency combined with the ascension of the sustainability rhetoric aligned with a greater focus on demand management and leakage reduction (POST 1995) seen as less wasteful and environmentally damaging than continually expanding supply.

Yet the new system has also been challenged as concerns have arisen around whether legal standards are appropriate, are being sufficiently enforced, and are being delivered in a way that is economically and politically acceptable to the public. Recently, investment into water resources including new supply infrastructure is back on the agenda due to recognition of climate change, resilience and the need to meet environmental protection standards (EA 2020). Pursuing both demand and supply strategies has been termed the “twin-track” approach.

### Planning Discourses

Planning is in a period where methods and frameworks are evolving to try and accommodate complex and contested concepts of risk, uncertainty, and value. The developments are happening quickly and simultaneously with limited coordination causing misalignments and inconsistencies, and sometimes the wrong or inappropriate tool being applied. Different actors tend to prefer different approaches, either more technical, more participatory, more legal, or simpler. It will be useful to build a framework that allows for innovation but also ensures suitable and clear assumptions to avoid plans becoming overly complicated, error-prone, opaque, inconsistent, or misunderstood.

#### Risk

A key boundary condition of water resources planning relates to establishing the risk appetite to act in light of water scarcity (Hall et al. 2012). However, risk is an elusive concept steeped in inherent subjectivity and uncertainty. What is considered an acceptable level of risk may be influenced by events such as droughts and funding barriers shaped by wider political and economic conditions. Clear planning assumptions are needed to allow for the development of plans on a consistent basis that can be held accountable. Recently multiple approaches to risk have developed and are in concurrent use including the 0.02% resilience standard, headroom, and emergency storage. This variation of approach introduces incompatibilities and confusion, particularly when guidance is undefined.

#### Uncertainty

Grappling with the challenge of uncertainty, particularly in the long term, planners are developing new, increasingly sophisticated methodologies to try and encompass a more robust attitude to risk with methods spanning use of stochastic forecasts, adaptive planning, scenario scanning and robust optimisation, and storyline approaches (Chan et al. 2022; Fowler and Kilsby 2007; Glenis et al. 2015; Hall et al. 2012).

#### Multiple Values (Trade Offs)

Investment decisions have long used cost-benefit analyses. Recently, regulatory guidance has introduced concepts such as “best value” planning, encouraging the use of multi-criteria decision-making methods that use a broader range of metrics beyond monetary cost, such as environmental and carbon metrics.

The evolving approaches have potential to better capture risk and value. The technical knowledge required for the complex planning primarily resides within water companies and consultancies which tend to favour quantitative methods. However, having a wide variety of modelling approaches may not fit within the constraints of collaborative planning frameworks. Furthermore, the increased sophistication and complexity of developing approaches make them vulnerable to accusations of being “black box” and difficulties understanding and communicating their outputs can hinder transparency and accountability. Moving towards more transparent and adaptive approaches to align with the needs of inherently uncertain and subjective “wicked problems” requires a shift in regulatory and company perspectives, likely causing disruption and requiring training. Furthermore, there are growing expectations for more participation and engagement with stakeholders and the public to better elicit their preferences in decision-making (Metcalfe and Sen 2022; Page and Bakker 2005). Successfully navigating this intricate landscape requires effective elicitation and facilitation techniques to integrate competing priorities.

### Rules

#### Siloed legal frameworks

Planning is characterised by its siloed nature in terms of legislation and policy guidance. Despite the interconnected nature of many policy areas such as water quality, the wider environment, spatial planning, agriculture, and energy, planning frameworks tend to be separate and constrained by legislation (Kidd and Shaw 2007). This can be beneficial for setting minimum requirements and maintaining accountability within the responsible authorities. Notable legislation has ensured the delivery of substantial investment to meet critical objectives, including (1) EU WFD stipulations for environmental protection and (2) the Water Industry Act 1991 which includes clauses ensuring protection of supply to customers. On the other hand, siloed legislation can also create barriers to a more comprehensive evaluation of trade-offs across intrinsically related agendas. More integrated planning is advocated as a solution to short-term and narrow decision making, however, the approach hasn’t been widely adopted in practice (Biswas 2008; Fritsch 2017). Yet, given the increasing uncertainty and potential for conflict, a more holistic approach to planning across water resources, as well as wider sectors could be beneficial as it could reflect the interconnected nature of issues such as climate change, growth, and the need for greater resilience.

#### Cost of capital

A critical part of water resources planning relates to the importance of finance and access to capital to pay for investment into sometimes large and costly infrastructure projects, shaped by regulatory and institutional arrangements. This is true today as well as historically, for example Hassan (1985, p.534) notes that in the 1800s “*another consideration in a period of rapidly growing water demand was [local authorities’] ability to raise capital for expanding works under the terms of their enabling legislation. By contrast “the facilities for long term borrowing. . were entirely lacking to the local authorities” in the early nineteenth century.*” (Hassan 1985, p. 534). Under privatisation, water companies use shareholder equity and borrow on capital markets based on their credit ratings to fund proposals for investments. All capital raised for investment plus interest and dividend payouts is ultimately paid for by customers’ bills over time. The framework has allowed companies flexibility in financial management with some companies choosing higher levels of gearing/debt than others. Greater debt can create financial pressures during times of economic instability and fluctuating interest rates. The additional costs of servicing debt placed on customers carries risks, particularly if seen to constrain proposals for investment. Ofwat must balance the risks around politically acceptable levels of debt, equity, and profit making against its duty under the Water Industry Act 1991 to make the sector attractive to capital investment and ensure the companies survive financially, (notably also serving to ensure privatisations success and safeguard its own role). These financial arrangements are relevant to water resources planning as investment may be constrained by the models of financing taken up by companies and regulated by Ofwat.

### Communities of Actors

#### The Government (and Executive)

Power ultimately rests with the government, including the prime minister, the cabinet, the treasury, the Secretary of State for Defra, Defra itself, and latterly the regulators. The government exercises power through approving final water resource management plans, through having the final decision in public inquiries, through producing policy and legislation, and through issuing top-down steer. However, despite maintaining a strong, central control on ultimate decision-making and overseeing the planning process, the government is distanced from the technical operation and function of the systems, devolving accountability away from the centre and placing responsibility for delivery onto the regulators and water companies (Watson et al. 2009). It is noteworthy that ministers are influenced by wider events such as upcoming elections, media stories, recent droughts, competing priorities, and internal political questions. These exogenous factors can potentially constrain or create opportunities for long term planning of water particularly regarding politically acceptable levels of investment and affordability.

#### The regulators

Ofwat and the EA operate within their distinct remits as defined in legislation, setting guidance, incentives, pricing, and issuing fines. Despite initiatives encouraging collaboration, their structures promote a compartmentalised approach due to their distinct responsibilities. Over time, the reach of regulatory guidance has grown considerably whereby planning frameworks such as the water resources planning guideline are increasingly prescriptive (Watson et al. 2009). They work closely with the government and water companies, leveraging their technical teams’ expertise on economic and environmental matters in the development and scrutiny stages of water company planning. They ultimately advise the government as to whether company plans should or should not be approved, based on their interpretation of policy and legislation, which contain a certain degree of flexibility. There is no specific regulator for the technical aspects of water resource planning, such as relating to defining methods for uncertainty and risk or approaches around demand management, leakage, and distribution networks. These responsibilities generally fall across regulators, but they rely heavily on the expertise of water companies and consultancies.

#### The water companies

Water companies are crucial actors within water resources planning as they are responsible for the technical planning, delivery and operation of their water networks. As natural monopolies, regulation is required to ensure companies meet their statutory obligations and policy guidance to deliver sufficient water to their customers. Companies are incentivised by both “carrot and stick” regulation approaches. Under the price cap system, they are able to recoup profits gained from the difference between expected and actual cost of delivery, i.e., profit maximisation by cost minimisation. Companies are motivated primarily to deliver profit under their fiduciary duties to their shareholders, with the exception of Welsh Water which operates as a non-profit (Bakker 2003; Owen 2013). If companies fail to meet the legal and expected minimum requirements, they will be held accountable by the government and regulators, either by the rejection of their proposed plan, or by penalties, fines, and theoretically licence withdrawal, though this has not happened in practice and is unlikely as licences have a 25-year notice period.

#### Public participation

Local public participation dropped in 1972 with the creation of RWAs and consequent severance between water planning and local authorities (Watson et al. 2009). Technocratic frameworks continued to develop establishing decision-making strongly dominated by the government and water authorities. With privatisation in 1989 the public were reframed as customers and as such have seen their representation grow through Ofwat’s duties to protect their rights as consumers. Ofwat sets rules and incentives for water companies (Franceys 2006), subject to influence from changing public expectations reported by the media, such as the increased attention on leakage following drought and hosepipe bans in 2020, seen as driving the introduction of higher leakage reduction targets. In this way the public can be seen to influence to the direction of water resources planning. Ofwat has also encouraged the elicitation and representation of customer views by water companies through “willingness to pay surveys” and other developing methods to justify that investments in water resources infrastructure reflect public value (Metcalfe and Sen 2022). Additionally, the statutory consumer protection body the Consumer Council for Water (CCWater) aims to represent the collective voice of consumers in policy debates (Franceys 2006), however, participation with CCWater may be considered secondary to consulting directly with Ofwat and water companies. Other forms of participation such as through lobbying by special interest groups continue as an important means of influence. For example, in 2010 the Group Against Reservoir Development (GARD) successfully campaigned against a new reservoir in Oxfordshire in the southeast of England during a public inquiry on the matter. More formal modes of consultation and public inquiry tend to reflect participation by already influential technical and strategic actors in water resources decision-making or “sanctioned” opposition groups, rather than necessarily the wider public (Kidd and Shaw 2007; Page and Bakker 2005). Finally, emerging forms of participation at regional, sub-regional, and catchment levels may become important as sites of engagement, particularly in places where local water allocation becomes more contested (Grecksch and Landström 2021).

## Conclusion

Water resources planning has changed from a piecemeal to a multi-scale approach. The recent transition sees new and evolving frameworks at national, regional, company, and sub-regional levels. These arose in response to growing recognition of the need to increase resilience in the face of climate change, growth, and environmental pressures, alongside recognition that the existing planning frameworks established at privatisation had gaps and limitations that would hinder efforts to plan more strategically and potentially gain consent for large new infrastructure assets, such as reservoirs. The shifting landscape illustrates the need for adaptive governance which allows frameworks that acknowledge the world as it is; uncertain and unstable, with inherent misalignments and fragmentation, and continually developing technical methods and innovations which have the potential to disrupt established systems. The shape of change will vary in each region at a particular time. It is therefore important to understand the specific local context and dynamics as they play out to be able to introduce reforms that are a good fit with a better likelihood of success.

We discuss key features of planning in the English and Welsh context. Firstly, these are fragmented, complex systems which cannot be addressed via a one-size-fits-all approach. Secondly, planning operates under a neoliberal backdrop, established since the 1980s, which emphasises economic efficiency. Thirdly, while concepts of risk appetite, uncertainty and value are inherently subjective, they tend to be managed using technical quantitative approaches, which have advantages for representing complex systems but can introduce challenges for transparency. Fourth, legislation is often siloed, although there are increasing calls for more integrated planning. Funding is a critical aspect, with access to capital determined by the institutional environment and wider economic and political forces. Additionally, ultimate power lies with the government and latterly with regulators. Companies are accountable for detailed planning and delivery, motivated by profit, and regulated by standards and reputational incentives. Finally, public participation is framed by consumer engagement.

Overall, there are some inherent tensions in the system whereby the government and regulators setting the rules lack detailed understanding of the systems, resulting in sometimes contradictory, misguided guidance, and the companies controlling the systems, who may be better placed to make decisions over how to improve their operation, are incentivised to meet statutory requirements to avoid penalties, maintain reputational standing and make efficiencies to generate profits, rather than directly deliver improvements for wider societal good. Key to the framework functioning is aligning incentives for public good with financial rewards and ensuring regulators are able to hold the companies to account to meet societies expectations as well as curb tendencies that do not align with the public good.

It is useful to outline the shifting landscape of water resources planning and highlight key features in light of the more strategic approach that has recently emerged. A new more collaborative model has been instigated with a multi-scale governance structure, including five new regional groups currently operating on a voluntary basis, instilling new requirements for alignment, cooperation, and resourcing. Further research could explore and evaluate the current arrangements and how they are evolving in order to recommend how reforms may best succeed, acknowledging existing constraints and opportunities such as those laid out in this paper.

## Data Availability

The data and materials supporting the findings of this study are available upon request. However, it is important to note that this paper does not involve the publication of a specific dataset. For inquiries regarding the data and materials used in this research, please contact A. Leonard.
